# Prevalence and associated factors of TB and HIV coinfections among adult inmates with presumptive pulmonary TB in a Kenyan prison

**DOI:** 10.1186/s41182-024-00623-2

**Published:** 2024-08-16

**Authors:** Suleiman Athuman Mwatenga, Ali A. Musa, Margaret W. Muturi, Abednego Moki Musyoki

**Affiliations:** 1 Department of Medical Laboratory Sciences, Moi County Referral Hospital, P.O. Box 18-80300, Voi, Kenya; 2 Clinical Laboratory, Msambweni County Referral Hospital, P.O. Box 8-80404, Msambweni, Kenya; 3 Department of Medical Laboratory Sciences, Kenyatta University, P.O. Box 43844-00100, Nairobi, Kenya

**Keywords:** Prevalence, TB and HIV, Coinfections, Associated factors, Prison

## Abstract

**Background:**

Tuberculosis (TB) is more than ten times higher in prisons compared to the general population, and HIV-infected persons are at increased risk of developing active TB and death. In the World Health Organization (WHO) African region, however, where the TB and HIV coinfections are highest, and prisons rarely factored in national disease surveillance, epidemiological data to inform TB control interventions in correctional facilities is limited. In this study, we assessed the prevalence of TB and HIV coinfections, as well as the factors associated with coinfections in our study setting.

**Methods:**

This was a prospective cross-sectional study among 157 adult (≥ 18 years) prisoners presenting with symptoms of pulmonary TB at Shimo La Tewa Prison, Kenya, between January and June 2023. The study excluded those with a history of anti-TB drugs use or on treatment follow-up and collected demographic and clinical characteristics data using a questionnaire. Sputum samples were collected and processed immediately using Xpert® MTB/RIF assay or stored at 4 °C for three (3) days in case of delay.

**Results:**

The overall prevalence of TB among inmates with presumptive pulmonary TB was 10.2%, 95% CI 6.37–16.91% (16/157), HIV 19.1%, 95% CI 13.73–25.97% (30/157). All the TB cases were positive for HIV (16/16, 100%), translating to TB/HIV coinfection of 10.2%, 95% CI 6.37–16.91% (16/157), and there was no rifampicin resistance. TB and HIV coinfection cases were found among underweight (100%, 16/16) prisoners*.* The independent factors associated with TB and HIV coinfections were education level (adjusted OR = 0.17, *p* = 0.007), smoking history (adjusted OR = 3.01, *p* = 0.009) and illegal drug use history (adjusted OR = 4.55, *p* = 0.044).

**Conclusion:**

We report a high prevalence of pulmonary TB and HIV coinfections among adult inmates with presumptive pulmonary TB in Kenya, with education level, smoking status, and illegal drug use as the independent factors associated with the coinfection. The authority should take measures to protect HIV-positive prisoners from TB, focusing on education, nutrition, smoking, and illegal drug use.

## Background

Tuberculosis (TB), caused by *Mycobacterium tuberculosis* (Mtb), remains the second leading infectious disease killer worldwide after Corona Virus Disease (COVID) 2019 [1]. Globally, there were an estimated 10.6 million TB cases and about 1.3 million deaths, including 167,000 people with human immunodeficiency virus (HIV) in 2022. The TB burden was disproportionately higher in the World Health Organization (WHO) South-East Asia (46%) and Africa (23%) regions, with Kenya in the global list of the 30 high-TB and TB and HIV burden countries [1].

In the face of the sluggish development of new TB vaccines, the emerging multidrug-resistant TB, and the rising global challenges, including COVID-19, energy and food insecurity, as well as the ongoing war in Ukraine and Gaza, multisectoral action to combat the TB pandemic remains a top global health priority [1]. TB epidemiology is attributable to comorbidities that impair immunity, such as diabetes and HIV infections, malnutrition, smoking (especially among men), household air pollution, poverty, and imprisonment [2].

Prisoners are reportedly at increased risk of contracting TB, with the burden being more than ten times higher than in the general population [3]. The global estimate of TB incidence in prisons is 15 cases per 100 person-years and is highest in the WHO African region (2190 cases per 100,000 person-years) [4]. In a recent systematic review and meta-analysis by Mera and others on the prevalence and predictors of pulmonary TB among prisoners in sub-Saharan Africa, the pooled pulmonary TB prevalence was 7.7% [2]. Further, HIV-infected inmates are at higher risk of developing TB than their HIV-negative counterparts [5]. HIV infection is the most significant risk factor for reactivating latent TB to active disease, and coinfection accelerates immunological function decline, increasing mortality in untreated cases [6]. Globally, the pooled prevalence of TB and HIV coinfections among prisoners ranges from 2.4 to 73.1% [5]. In Africa, few studies have documented TB and HIV coinfections, with prevalence ranging from 1.13 to 58 per cent, in prisons. These studies were conducted in Ethiopia [7, 8], Zambia [9, 10], Nigeria [11, 12], Cameroon [13, 14], Tanzania [15] and South Africa [16–18], Uganda [18]. There is limited data on TB and HIV coinfections in Kenyan prisons [19].

The high TB and HIV burden among prisoners is attributable to the lack of quality healthcare, such as TB diagnostic services and active case screening, occasioned by low priorities by policymakers, poor infrastructural designs that favour inadequate ventilation and overcrowding, undernutrition, HIV infection, alcohol use disorders and smoking [2, 3]. The World Health Assembly in 2014 adopted the WHO End TB strategy as part of the newly established Sustainable Development Goals (SDGs) to attain a 90% reduction in the TB incidence rate and a 95% reduction in the absolute number of TB deaths by 2035, with priorities on TB Key populations, including refugees, the poor, refugees, people living with HIV, and prisoners [1].

Inmates can serve as reservoirs for Mtb transmission within the prison and the communities after their release [2, 20]; as such, there is a need for systematic and continuous surveillance to curb the spread of infections, especially those caused by multidrug-resistant strains, through early case detection and treatment initiation. In many resource-constrained settings, such as Kenya, prisons are often neglected and excluded from the national health statistics [21]; therefore, epidemiological data to inform TB infection prevention interventions are limited. In this study, we assessed the prevalence of TB and HIV coinfections, as well as the factors associated with coinfections among adult (aged 18 years or more) inmates with presumptive pulmonary TB in a Kenyan prison.

## Materials and methods

### Study area, design, and population

We adopted a prospective cross-sectional study at Shimo-La Tewa Health Centre, a 24-bed facility that serves inmates from Shimo Latewa Maximum Security Prison, located along Malindi Road in Shanzu, Mombasa County, Kenya. The prison has a capacity of 1500 inmates drawn from all over the country. All adult (aged ≥ 18 years) inmates presenting with signs and symptoms suggestive of TB (such as prolonged cough with or without blood, chest pain, fatigue, weight loss, fever, or night sweats) at the Shimo la Tewa prison chest clinic were eligible for enrolment. The study participants were recruited from January to June 2023, excluding those with a history of anti-TB drug use or on treatment follow-up, as well as those who declined to participate.

### Samples collection

This study collected data on participants’ demographic and clinical characteristics using a pretested structured questionnaire administered through face-to-face interviews. All participants were requested to provide sputum (early morning and spot) samples in 2 consecutive days. We instructed the patients to rinse their mouth twice with water, inhale deeply, cough vigorously, and expectorate into a clean and sterile 50-ml falcon tube. The samples were received and processed immediately. In case of delay, samples were stored at 4 °C and processed within 3 days [7].

### Laboratory analysis

The sputum samples were screened for TB using Xpert® MTB/RIF (Cepheid, Sunnyvale, CA, USA), following the manufacturer’s instructions. Compared to culture, the sensitivity and specificity of Xpert MTB/RIF are reported at 85% and 98%, respectively [22]. Briefly, 0.5 ml of sputum sediments were resuspended with 1.5 ml Xpert/RIF sample reagent, thoroughly shaken 15 times, and incubated for 10 min at room temperature. After incubation, we thoroughly shook the mixture and re-incubated it for 5 min. The liquefied sample was transferred into an Xpert MTB/RIF cartridge labelled with the patient ID, loaded in Xpert MTB/RIF, run for one hour, and read the results from the machine-generated printouts.

After counselling by a professional counsellor, patients were screened for HIV using Determine™ HIV-1/2 Kits (Abbott Laboratories, USA), with positive samples confirmed by First Response HIV-1–2 (Premier Medical Corporation Ltd., Kachigam, India) per the Kenya Ministry of Health algorithm. The Determine™ HIV-1/2 Kit has a sensitivity of 100% and a specificity higher than 99% [23], while First Response HIV-1–2 has a sensitivity and specificity of 100% [24].

### Data analysis

The data were coded and entered into password-protected MS Excel 2013, checked for completeness, and exported and analysed in Epi-info 7, with categorical data presented in tables as frequencies and percentages and continuous data as median and interquartile range. We investigated the normality for age using the Shapiro–Wilk test and obtained a *p*-value < 0.001, indicating a non-normal distribution. We used Stata 17 to calculate 95% Confidence Intervals (CI) for proportion. After assessing the collinearity and interaction of variables, we developed bivariate models to explore the association between TB and HIV coinfections and patients’ demographic and clinical characteristics, with associations with *p*-values ≤ 0.2 subjected to a multivariable analysis using binary logistic regression. Interactions between education level, smoking status, and illegal drug use to determine their significance and potential impact on the study outcome were assessed, with variables showing significant interaction (smoking and drug use) subjected to a multivariable model and those with non-significant interaction removed to maintain model parsimony. We checked for multicollinearity using the Variance Inflation Factor (VIF), with education level (VIF = 1.07), use of illegal drugs (VIF = 1.06), and drug use (VIF = 1.01) showing low to moderate (1 < VIF < 5) multicollinearity, which is typically acceptable [25]. The statistical significance level was at *p* < 0.05 [95% confidence interval (95% CI)], and statistically significant associations are bolded in Table 2.

## Results

### Demographic and clinical characteristics of study participants

Of 165 adult (aged ≥ 18 years) inmates presenting with signs and symptoms suggestive of TB at the Shimo la Tewa prison chest clinic, we excluded 4.8% (8/165) because they declined to grant the study informed consent (75%, 6/8) and had previous TB history (25%, 2/8). In total, we recruited 157 participants, with a median age of 37 (interquartile range [IQR]: 30–46.5) years, and predominated by males (89.8%) aged 31 to 41 years, and those with normal BMI (62.4%) (Table 1).

**Table 1:** Demographic and clinical features of study participants

Characteristics	Frequency (%)
Gender	
Male	141 (89.8)
Female	16 (10.2)
Age (years)	
18–29	32 (20.4)
30–44	80 (51.0)
≥ 45	45 (28.7)
BMI	
Underweight (< 18.5)	41 (26.1)
Normal weight (18.5–24.9)	98 (62.4)
Overweight (25.0–29.9)	18 (11.5)
Education level	
Secondary	58 (36.9)
Primary	73 (46.5)
None	26 (16.6)
Pre-existing health condition	
Diabetes	12 (7.6)
Hypertension	11(7.0)
Smoking	
Yes	64 (40.8)
No	93 (59.2)
Smoking duration ( in years)	
< 10	8 (5.1)
11–19	26 (16.6)
≥ 20	29 (18.5)
Alcohol use	
Yes	40 (25.5)
No	117 (74.5)
Alcohol duration (in years)	
≤ 10	8 (20.0)
11–19	15 (37.5)
≥ 20	17 (42.5)
Use illegal drugs	
Yes	14 (8.9)
No	143 (91.1)
TB clinical symptoms	
Cough	157 (100.0)
Fever	34 (21.7)
Chest pain	14 (8.9)
Weight loss	16 (10.2)
Night sweat	122 (77.7)

HIV: human immunodeficiency virus; BMI: body mass index; %: per cent

### Prevalence of TB and HIV coinfections

**Fig. 1:**
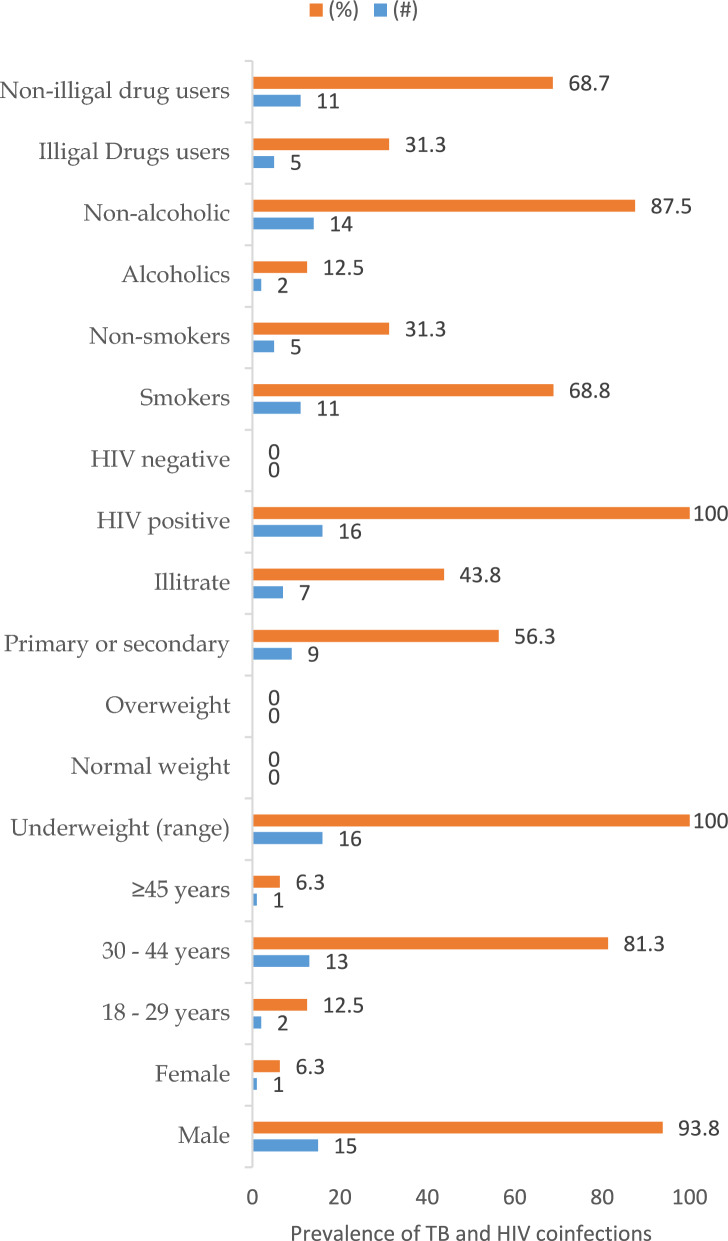
Prevalence of TB and HIV coinfections: %: percentage; #: number of cases; HIV: human immunodeficiency virus

The overall prevalence of TB among the study participants with presumptive pulmonary TB was 10.2%, 95% CI 6.37–16.91% (16/157), HIV 19.1%, 95% CI 13.73–25.97% (30/157). All TB cases were positive for HIV (16/16, 100%), translating to TB/HIV coinfection of 10.2% 95% CI 6.37–16.91% (16/157), and there was no rifampicin resistance. The TB and HIV coinfection prevalence was highest among prisoners who were underweight (100%, 16/16) and with a smoking history [68.8%, 95% CI 66.04–91.46% (11/16)] (Fig. 1).

### Factors associated with TB and HIV coinfections

The study bivariate analysis showed that education level, smoking, and use of illegal drugs were significantly associated with TB and HIV coinfections among the prisoners (Table 2). Participants aged ≥ 45 years were 88% likely to have a TB and HIV coinfection compared to those aged between 18 and 29 years [crude odds ratio (OR) = 0.12, 95% confidence interval (CI) 0.02–0.93, *p* = 0.042]; however, this was not an independent association [adjusted OR = 0.15, 95% CI 0.02–1.25, *p* = 0.079].

**Table 2:** Factors associated with TB and HIV coinfections

Variables	TB and HIV coinfections		Crude OR (95% CI)	*P*-value	Adjusted OR (95% CI)	*P*-value
	Present frequency (%)	Absent frequency (%)				
Gender						
Male	15 (93.8)	126 (89.4)	1.79 (0.22–14.93)	0.495	¶	
Female	1 (6.3)	15 (10.6)	Ref			
Age (years)						
18–29	2 (12.5)	30 (21.3)	Ref		Ref	
30–44	13 (81.3)	67 (47.5)	0.34 (0.03–3.93)	0.388	0.58 (0.04–7.64)	0.677
≥ 45	1 (6.3)	44 (31.2)	0.12 (0.02–0.93)	**0.042**	0.15 (0.02–1.25)	0.079
BMI						
Underweight (< 18.5)	16 (100)	25 (17.7)				
Normal weight (18.5–24.9)	0	98 (69.5)				
Overweight (25.0–29.9)	0	18 (12.8)				
Education level						
Primary or secondary	9 (56.3)	122 (86.5)	0.20 (0.07–0.60)	**0.006**	0.17 (0.05–0.61)	**0.007**
None	7 (43.8)	19 (13.5)	Ref			
HIV status						
Positive	16 (100)	14 (9.9)				
Negative	0	127 (90.1)				
Diabetes						
Yes	0	12 (8.5)				
No	16 (100)	129 (91.5)				
Hypertension						
Yes	1 (6.3)	10 (7.1)	0.87 (0.10–7.30)	0.689	¶	
No	15 (93.8)	131 (92.9)	Ref			
Smoking status						
Yes	11 (68.8)	53 (37.6)	3.65 (1.20–11.09)	**0.029**	3.01 (1.83–10.94)	**0.009**
No	5 (31.3)	88 (62.4)	Ref		Ref	
Smoking (years)						
< 10	1 (7.7)	7 (14.0)	Ref			
11–19	6 (46.2)	20 (40.0)	1.83 (0.19–17.85)	0.605	¶	
≥ 20	6 (46.2)	23 (46.0)	0.87 (0.24–3.13)	0.831	¶	
Alcohol consumption						
Yes	2 (12.5)	38 (27.0)	0.39 (0.08–1.78)	0.362		
No	14 (87.5)	103 (73.0)	Ref			
Alcohol use (years)						
≤ 10	1 (7.7)	7 (14.0)	Ref			
11–19	6 (46.2)	20 (40.0)	1.83 (0.19–17.85)	0.605	¶	
≥ 20	6 (46.2)	23 (46.0)	0.87 (0.24–3.13)	0.831	¶	
Use of illegal drugs						
Yes	5 (31.3)	9 (6.4)	6.67 (1.90–23.37)	**0.007**	4.55 (1.04–19.81)	**0.044**
No	11 (68.8)	132 (93.6)	Ref			

*OR* odds ratio, *CI* confidence interval, *BMI* body mass index

^¶^Binary logistic regression was not computed because crude OR *p*-values were greater than 0.2

Multivariate analysis established that those with primary or secondary education were 83–85% less likely to have TB and HIV coinfections (adjusted OR = 0.17, 95% CI 0.05–0.61, *p* = 0.007) when compared with those having no formal education. Participants with a smoking history were three times more likely to have TB and HIV coinfections (adjusted OR = 3.01, 95% CI 1.83–10.94, *p* = 0.009) when compared with those with the history. Further, prisoners with an illegal drug use history were five times more likely to have TB and HIV coinfections (adjusted OR = 4.55, 95% CI 1.04–19.81, *p* = 0.044) compared to those without such a history (Table 2).

## Discussion

In this study, the prevalence of tuberculosis (TB) among prisoners presenting with presumptive pulmonary TB was 10.2%, higher than reported elsewhere in Ethiopia (2.8–8%) [7, 26, 27], Uganda (2.02%) [28], South Africa (2.7–3.5%) [29, 30], Brazil (3.9%) [31], and in Central Region of China (1.2%) [32] prisons. TB prevalence in the current study was 33 times higher than reported in the general population by the latest Kenyan National TB Prevalence Survey of 2016 [21]. Our finding is consistent with other reports that TB incidence is 5 to 70 times greater in prisons than in communities [33, 34]. Inmates with TB can serve as hotspots for TB transmission within prison walls and outside communities after their release [20], highlighting the public health importance of interventions targeting this key population in national and global efforts to eradicate TB. The high TB burden in prisons is attributable to low health services priorities by public health policymakers, infrastructural designs that favour overcrowding with no sufficient ventilation, and prisoners’ vulnerability due to malnutrition, HIV infection, smoking and alcohol use disorders [3].

In our study, all the TB cases were positive for HIV (16/16, 100%), with a higher HIV prevalence (19.1%) compared to the national prevalence (5.9%) in Kenya [35]. The TB/HIV coinfection of 10.2%, in the current study, was higher than reported in East Gojjam Zone (1.13%) [7] and Gondar Zone (3.6%) [8] in Northwest Ethiopia, Zambia (1 to 6.4%) [9, 10, 36], and Nigeria (4.2%) [11], but lower than documented in Tanzania (25.9%) [15], Uganda (57.1%) [18], Nigeria (24.4%) [12], Cameroon (25%) [14], and South Africa (46.6 to 58%) [16, 17, 37]. A global systematic review by Edge and others (2016) on prisoners coinfected with TB and HIV estimated a coinfection prevalence ranging from 2.4 to 73.1% [5]. There exists a significant association between HIV and TB [7], as well as a patient’s history of prison and TB/HIV coinfection [38]. Notably, prisoners infected with HIV are at high risk of developing TB, but the magnitude of the risk varies between different prisons and countries [5]. Therefore, local epidemiological data on TB and HIV coinfections in prisons remain critical to guide infection prevention intervention in line with the World Health Organization (WHO) ‘End TB Strategy’, endorsed by the Sixty-seventh World Health Assembly in 2014 [1]. This strategy is part of the newly established SDGs to attain a 90% reduction in the TB incidence rate and a 95% reduction in the absolute number of TB deaths by 2035, with priorities on TB Key populations, including prisoners [1].

The TB and HIV coinfection cases were highest among prisoners who were underweight (100%, 16/16) and with a smoking history (68.8%, 11/16), which are considered risk factors for the acquisition of TB [9, 39]. Further, participants who had primary or secondary education were 83–85% less likely to have TB and HIV coinfections (adjusted OR = 0.17, 95% CI 0.05–0.61, *p* = 0.007) when compared with those having no formal education. Our finding corroborates that of previous studies in prisons by Valença and colleagues in southern Brazil [40], Biadglegne and others in Northern Ethiopia [41], and Shimeles and colleagues in the Ethiopian general population [42], where illiterate persons were more than twice as likely to develop TB compared to those who could at least read and write [40]. Education improves knowledge, skills, reasoning, effectiveness, and a broad range of other abilities that can improve health [43], suggesting a need for health education interventions to alleviate the TB burden in the current study settings and beyond.

In the current study, prisoners with a history of illegal drug use were five times more likely to harbour TB and HIV coinfections (adjusted OR = 4.55, 95% CI 1.04–19.81, *p* = 0.044) compared to those without such a history. A study by Arroyave and colleagues showed a similar association where Prison Guards with a history of drug use at least once in a lifetime were two times at risk of latent TB [44]. Similarly, in Southern Brazil, the burden of TB was highest among prisoners with a history of illegal drug use [45]. Drug users are more likely to be infectious and take longer to achieve negative culture, with physiological effects of drug use, such as direct impairment of the cell-mediated immune responses by opiates, along with the environment and risk behaviours of drug users likely playing a critical role in TB epidemiology among drug users [46].

Our study findings showed that prisoners with a smoking history were three times more likely to have a TB and HIV coinfection (adjusted OR = 3.01, 95% CI 1.83–10.94, *p* = 0.009) when compared with those without such a history. A similar association was documented in Ethiopia [47], South Africa [30, 37], southern Brazil [40], and Pakistan [48] prisons. Smoking is a contributor to the high TB burden in prisons, for it increases the risk of infection, developing the active form of the disease and ultimately dying from it, negatively influencing the response to treatment and increasing the risk of relapse [1, 49].

## Conclusion

We report a high prevalence of pulmonary TB and HIV coinfections among adult inmates with presumptive pulmonary TB in Kenya, with education level, smoking status, and illegal drug use as the independent factors associated with the coinfections. The authorities should take measures to protect HIV-positive prisoners from TB, focusing on education, nutrition, smoking, and illegal drug use. Multicentric studies with large sample sizes are needed to substantiate the findings further.

## Study limitations

Despite being a single-centre study with a small sample size, which may limit the generalization of the findings in similar settings, our research offers insight into the significant burden of TB and HIV coinfections in prisons, particularly in resource-constrained countries where national health surveys often exclude correctional facilities.

## Data Availability

The datasets used and/or analysed during the current study are available from the corresponding author on request.

## Abbreviations

TB: Tuberculosis
MDR: Multidrug resistance
Mtb: *Mycobacterium tuberculosis*
HIV: Human immunodeficiency virus
WHO: World Health Organization
COVID-19: Corona Virus Disease 2019
DRC: Democratic Republic of Congo
SDGs: Sustainable Development Goals
